# Posterior Cruciate Ligament Tibial Avulsion treated with Open Reduction and Internal Fixation through the Burks and Schaffer Approach

**DOI:** 10.5704/MOJ.1507.004

**Published:** 2015-07

**Authors:** K Khatri, V Sharma, D Lakhotia, R Bhalla, K Farooque

**Affiliations:** Department of Orthopaedics, GGS Medical College, Faridkot, India; * Department of Orthopaedics, All India Institute of Medical Sciences, New Delhi, India; ** Department of Orthopaedics, Guro Hospital, Korean University Medical Centre, Seoul, Korea; *** Department of Orthopaedics, Orthotrauma Centre, New Delhi, India

**Keywords:** Joint instability, posterior cruciate ligament, rehabilitation, functional outcome

## Abstract

Objective: To report functional outcome in Posterior Cruciate Ligament (PCL) tibial avulsion fractures treated with open reduction and internal fixation through Burks and Schaffer approach. The patient specific functional outcome measures like IKDC grading together with objective grading with stress radiographs have rarely been used -to assess PCL tibial avulsion fractures.

Material and Methods: Twenty seven patients (21 males and 6 females) were included in the study. The mean follow up duration was 22.30±6.82 months. They were assessed using international knee documentation committee (IKDC) grades, Lysholm scoring and stress radiography. The injury severity scores (ISS) of the patients were also recorded.

Results: The mean Lysholm scores at the time of last follow up was 90.85±5.58. The IKDC grades achieved were normal in 20 patients, near normal in five and abnormal in two. The PCL laxity determined on active hamstring contraction stress radiography was grade I in 20 cases and grade II in seven cases. All patients had achieved bony union of tibial avulsion fractures at the time of last follow up. Statistically significant association was found between higher ISS and lower Lysholm scores. (t=3.455, p=0.0019). Good IKDC grades were associated with higher Lysholm scores (analysis of variance, F=32.51, p<.0001). There was no correlation between PCL laxity and functional outcome (t=.857, p = 0.399).

Conclusion: PCL tibial avulsion fractures treated through Burk and Schaffer approach with open reduction and internal fixation produces good results. The early rehabilitation without cast immobilisation prevents arthrofibrosis.

## Introduction

Posterior cruciate ligament (PCL) is the stronger of the two cruciate ligaments of knee and is a constraint to posterior dislocation of the knee in 90 degrees of flexion^1^. Damage to PCL either due to bony avulsion or intrasubstance rupture if not treated can lead to chronic pain and patellar degeneration due to posterior subluxation of tibia^2,3^. There is a difference between bony avulsion fractures and intrasubstance tear in terms of easy diagnosis on standard radiographs and widely accepted treatment protocol regarding its fixation^4^. The treatment of tibial bony avulsion may vary from open reduction and internal fixation to arthroscopic fixation with screws or sutures^5-7^.

The purpose of this study was to objectively evaluate the functional results after open reduction and internal fixation of tibial PCL avulsion fractures with international knee documentation committee (IKDC) grades and stress radiography. We hypothesize that the patients will report stable knees as assessed with stress radiographs, good range of motion and functional results as indicated by (IKDC) grades^8^ and Lysholm score^9^.

## Materials and Methods

The mechanism of injury was motor vehicle accident in 23 patients, fall from height in three and sports related injury (while playing kabadi- a rural sport) in one patient (table I). The age of the patients ranged from 21 to 62 years with mean age of 35.88±11.15 years. There was involvement of right side in twenty cases while the number was seven on the left side. Majority of the cases were treated within 10 days however in five cases there was delay of more than 10 days. In two cases, there was associated head injury and definitive fixation was staged.

**Table I:** Patient characteristics

Age (in years)	35.88±11.15
Gender (male: female)	21:6
Mechanism of injury	23:3:1
(MVA: fall : sports injury)	
Side involved (right : left)	20:7

The study was conducted with the approval of ethics committee of the institution and was performed according to the ethical standards of the 1964 Declaration of Helsinki as revised in 2000. Over the course of four years (2009 to 2012), 51 patients with PCL tibial avulsion were treated with open reduction and internal fixation at level 1 trauma centre. The patients treated with other methods or lost to follow up were excluded from the project. The hospital records were studied to determine the mode of injury, demographic data, injury severity score, delay in surgery, treatment given, complications of either the fracture or treatment and revision surgery if any required. The data regarding comorbid conditions, associated limb injuries and side of injury were also collected.

The ligament avulsion fractures were confirmed radiologically and patients were examined clinically by the senior authors. The patients were subjected to the Lachman test, and the anterior and posterior drawer test for integrity of cruciate ligaments. The collateral ligaments were assessed with varus and valgus stress in extension and in 30 degrees flexion. The Dial test and external recurvatum test were performed to assess the associated posterolateral ligament complex insufficiency. Magnetic resonance imaging (MRI) was carried out in all the patients prior to surgical intervention to look for associated bony and capsule-ligamentous injuries which could have been missed at initial evaluation or in case of inconclusive clinical examination findings. All the patients were examined again under anaesthesia in the operative room prior to surgical intervention.

### Operative technique:

Preoperative antibiotic (1.5 gm cefuroxime, intravenous) was administered in all the cases after sensitivity testing, one hour prior to skin incision, as a single dose. The operative procedures were performed under general anaesthesia with tourniquet control.

**Fig. 1:**
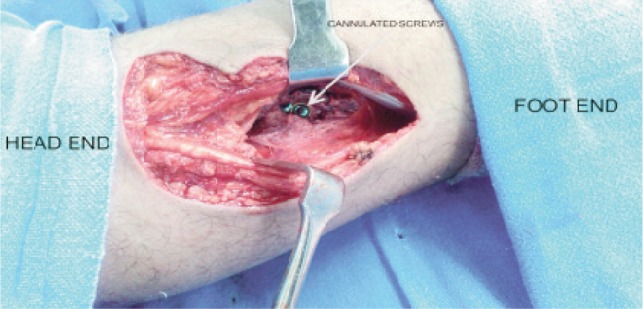
Image showing fixation of posterior cruciate bony avulsion injury with partially threaded cannulated cancellous screws.

**Fig. 2:**
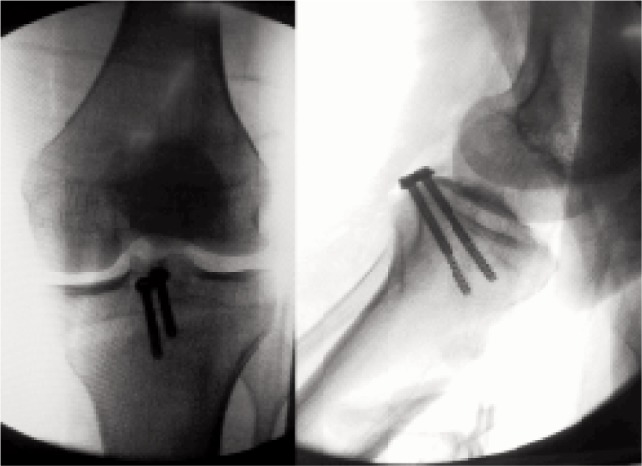
Flouroscopic image showing acceptable reduction of the avulsed posterior cruciate bony fragment.

The patients were positioned in prone position. Skin incision was made over the posterior aspect of knee with the horizontal limb over the popliteal crease and vertical limb on the medial aspect of gastrocnemius. The deep fascia over the medial gastrocnemius was incised and interval between medial gastrocnemius and semimembranosus tendon was identified. The dissection was carried bluntly with finger until the posterior capsule of knee joint was reached. The middle geniculate artery was ligated wherever necessary. The motor branch of the tibial nerve to medial head of gastrocnemius was preserved. The medial gastrocnemius was retracted laterally, thus protecting the neurovascular structures. The posterior aspect of femoral condyles and proximal tibia could be palpated at this stage. Slight knee flexion was done in almost all the cases for better visualisation. Recession of tendinous origin of medial gastrocnemius was carried out wherever necessary for enhancing exposure. The posterior knee joint capsule was incised vertically to access the contents of posterior intercondylar notch and tibial attachment of posterior cruciate ligament. The bony base of avulsion was debrided wherever necessary. The bony fragment was pushed down and secured with a kirschner’s wire and positioning verified under fluoroscope. The bony fragment was then fixed with 4.5/3.5 mm partially threaded screws (one or two, AO, Synthes) depending upon the fragment size (Figure 1). The position of bony fragment was again assessed under fluoroscope (Figure 2) and if found adequate the wound was washed and closed without drain.

Postoperatively, the patients were advised to wear hinged knee brace and ranges of motion exercises of the operated knee were started at 0 to 30 degrees from day one with the help of continuous passive motion machine. The range of motion of the operated knee was advanced as per pain tolerability and considering other associated injuries. The patients were allowed to bear weight as tolerated with knee brace locked in extension depending upon the concomitant injuries and advised to remove the brace for range of motion exercises. They were instructed to undergo rehabilitation under the guidance of a physical therapist and therapy was carried out twice a week at the institution for range of motion exercises, mobility and quadriceps strengthening. The active hamstring exercises were not allowed for eight weeks.

The patients were followed up at three weeks, six weeks, three months, six months and one year. Except at the first visit, in which only range of motion and local wound condition was addressed, subsequent visits included thorough clinical and radiological assessment. Clinical examination included posterior drawer test and radiological assessment was done with anteroposterior and lateral radiographs of knee. The patients were allowed to fully bear weight depending upon the associated injuries and ambulate without brace subsequent to bony union. Bony union was defined as bony consolidation seen on radiographs, absence of pain and stable knee. This was achieved in the majority of cases between ten to twelve weeks. Strengthening exercises were carried out after bony union and majority of the patients had returned to their previous occupation within six months.

The functional outcome in our study was assessed with clinical and radiological parameters like range of motion, ligament laxity, crepitus, subjective measurement, radiographic findings, activity levels and functional strength. The results were evaluated using international knee documentation committee (IKDC) grades and Lysholm scoring. The IKDC grading measures both signs and symptoms. Each parameter in IKDC grading is assigned an overall grade of A signifying normal, B denoting near normal, C denoting abnormal or D signifying severely abnormal^8^. The final grades are determined by lowest score obtained in each parameter.

### Statistical analysis:

The student’s t test (unpaired two-tailed) and analysis of variance (ANOVA) was used for statistical analysis. Statistical significance was set at a P-value of <0.05.

## Results

**Fig. 3:**
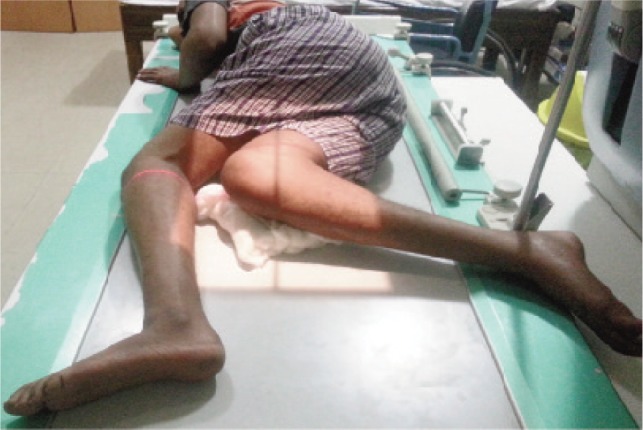
Patient postioning in stress radiography (active hamstring contraction test).

**Fig. 4:**
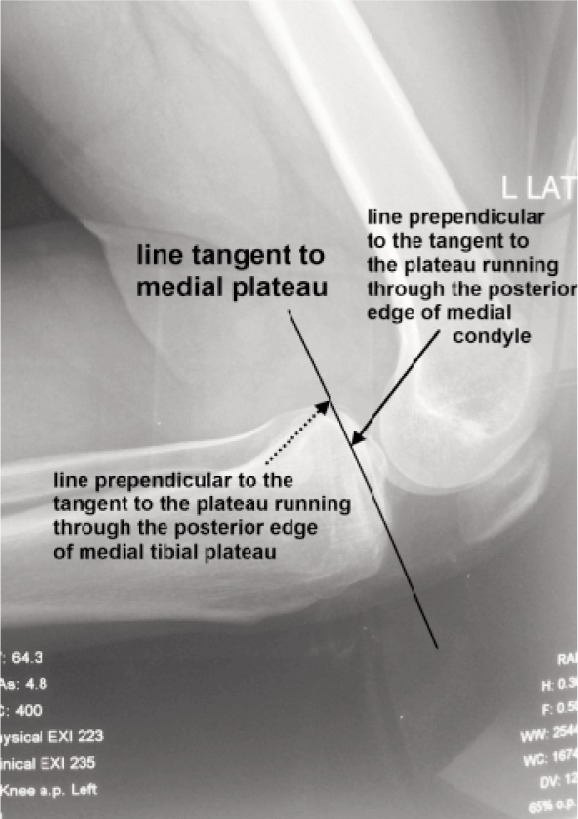
Stress radiograph to asses the posterior translation of the tibial plateau: a line is drawn tangent to the medial tibial plateau. Next a line is drawn prependicular to the tangent to the medial tibial plateau and passing through the posterior edge of medial tibial plateau. Subsequently, another line is drawn passing throught the posterior aspect of medial femoral condyle. The posterior translation is then calculated by measuring the distance between the two prependiculars drawn.

In the month of August 2014, an effort was made to review all the cases of PCL tibial avulsion treated with open reduction and internal fixation at the institute. The contact details of patients were searched in computerised patient record system of the institute. A total of 51 patients had been treated with open reduction and internal fixation during the period of four years (2009 to 2012). The patients were contacted over telephone and through postal address present in the institute’s database. Despite all efforts, thirty six patients could be contacted and among them thirty individuals consented for the study. Fifteen patients could not be traced. Finally twenty seven patients turned up for an additional hospital visit. They were asked to fill IKDC and Lysholm questionnaire. They were also subjected to clinical and radiological examination. The clinical examination was performed by the senior authors while IKDC and Lysholm scoring was performed by the other authors. Range of motion was recorded using standard goniometer and integrity of posterior cruciate ligament (PCL) was assessed by posterior drawer test and radiologically. The PCL laxity recorded on radiological examination was considered for final evaluation. They were also subjected to stress radiograph of both knees with active hamstring contraction as described by Chassaing *et al.*^10^ The patient was turned to lateral position with knee flexed to 90 degrees with heel supported. The patient was then asked to contract the hamstring for at least 10 seconds and the radiograph was taken (Figure 3). The posterior translation of medial compartment was measured in relation to the line drawn tangent to the medial plateau as described by Jacobsen et al11 (Figure 4). The PCL laxity was graded as grade I in case the laxity was less than 5mm, grade II with laxity ranging between 6 to 10 mm and grade III in cases with laxity more than 10 mm. There were 20 cases with grade II PCL laxity, seven cases with grade I laxity and none with grade III laxity.

The mean period of follow up was 22.30 ± 6.82 months. The mean Lysholm score was 90.85±5.58 in 27 patients (range 77 to 97). The IKDC grading was normal in 20 patients, near normal in five and abnormal in two patients at the final evaluation (table II). The average injury severity score (ISS) was 10.03±7.95 (range 4 to 35). The bony union was achieved in all the patients at the time of last follow up. The associated injuries (bony and ligamentous injuries) were treated in single stage or PCL bony avulsion was treated at a later stage. No patient had to undergo implant removal for hardware related problems.

**Table II:** International Knee Documentation Committee (IKDC) scores for the 27 patients

Parameter	Normal (Grade A)	Near normal (Grade B)	Abnormal (Grade C)	Severely abnormal (Grade D)
Subjective measure	21	4	2	0
Symptoms	19	6	2	0
Range of motion	24	2	1	0
Crepitus	23	4	0	0
Ligament laxity	20	7	0	0
Radiography	21	6	0	0
Functional testing	20	5	2	0
Final evaluation	20	5	2	0

### Correlation with findings:

No statistically significant correlation was seen between Lysholm scores and PCL laxity grading (t=.857, p = 0.399). The patients were divided in two groups with delay in surgery of duration less than 10 days and those with delay of equal or more than 10 days. There was no statistically significant correlation found between delay in surgery and final Lysholm scores (t=1.659, p= 0.109).

There was significant correlation between Lysholm scores and IKDC grades on Analysis of variance (ANOVA) testing. The higher IKDC grades were associated with good Lysholm score (ANOVA, F=32.51, p<.0001).

The patients were also divided into two groups based upon injury severity score (ISS) as those with score less than 11 and those with score equal to or more than 1112. A statistically significant association was found between ISS score equal to or greater than eleven and lower Lysholm scores (t=3.455, p=0.0019).

### Complications:

Two patients had developed arthrofibrosis. One patient was a 29 years old female who had sustained subarachnoid haemorrhage with knee dislocation at the initial presentation. The knee dislocation was reduced in the emergency department and above knee POP slab was applied. There was no distal neurovascular deficit. The patient was under intensive care in neurosurgery department and was taken for PCL avulsion fixation and ACL reconstruction after a delay of 28 days. At 10 weeks post operative, her knee flexion was limited to 70 degrees and manipulation under anaesthesia resulted in increase in range of motion to 120 degree. Patient had maintained range of motion from zero degree extension to 112 degrees of flexion at the time of last follow up at 29 months. Another patient was a 21-year-old male who had sustained motor vehicle injury and presented with extradural haemorrhage (EDH) and grade 2b open right femur fracture. The patient was taken up for EDH drainage and external fixator was applied for the compound femur fracture. The patient was taken for next surgery after a delay of 30 days and interlocking intramedullary nailing of femur along with fixation of PCL avulsion were performed. At eight weeks post operative- patient had 80 degrees of left knee flexion and he was advised to undergo manipulation of left knee under anaesthesia. Following manipulation the range of motion increased up to 130 degrees. Patient maintained 123 degrees of flexion at the time of last follow up at 15 months.

## Discussion

Various surgical approaches have been described for the fixation of PCL avulsion fractures. The standard classical approach described by Abbott^13^ was time consuming, as it required handling of popliteal vessels. Later on modifications were described by Trickey *et al*^14^, Ogata^-15^ and Burks and Schaffer^-16^ to the standard posterior approach. Trickey^-14^ had made some headway in decreasing the operative time but still required the division of the medial head of gastrocnemius which led to delayed rehabilitation. Ogata^15^ had described the osteotomy of fibular neck which increased the complexity of the procedure. Burks and Schaffer^16^ simplified the procedure and did not require division of medial head of gastrocnemius or fibular osteotomy. The post-operative rehabilitation was accelerated and good functional results were obtained. We had used this approach in the management of all the cases of PCL tibial avulsion injury.

Many authors had recommended immobilisation in cast for six weeks in cases of PCL avulsion treated by open reduction. They had reported stiffness as a major complication in their studies^14,17,18^. Nicandri *et al*^12^ had reported arthrofibrosis in only one of the ten cases when aggressive physiotherapy protocol was initiated instead of cast immobilisation. They recommended the use of functional brace and early range of motion exercises to achieve good functional results. However the prerequisite for the same is stable fixation. We followed the physiotherapy protocol as described by Nicandri *et al*^12^ and experienced arthrofibrosis in only two of the 27 patients.

There is controversy regarding the operative and non-operative management of intra substance tear of posterior cruciate ligament^19-21^, however there is consensus regarding the treatment of PCL tibial avulsions^17, 22^. Seitz *et al*^18^ had reported excellent results in their series of 26 patients treated for PCL bony avulsion with open reduction and internal fixation. Meyer^2^ had reported poor functional outcome in patients of bony PCL avulsion treated non-operatively. Earlier studies had used functional assessment tools like musculoskeletal functional assessment (MFA), Gillquist and Lysholm scores for the evaluation of functional results in PCL bony avulsion injuries^23-24^. Very few studies had evaluated the PCL avulsion injuries with assessment measures like IKDC scoring^25^ and stress radiography.

Musculoskeletal functional assessment (MFA) though a validated functional assessment tool which assesses the general well-being of the patient is not specific for knee function^12^. IKDC scoring assess the final outcome in PCL avulsion injuries in a more comprehensive manner than MFA.

Mazda *et al*^26^ had shown in their study that IKDC is more sensitive than Lysholm scoring for evaluation of ligament injuries. Singla *et al*^27^ had also propagated - the use of IKDC over Lysholm score for the assessment of functional results in PCL injuries. One possible reason for it could be that IKDC allows for more detailed assessment of signs and symptoms in comparison to Lysholm scoring. The Lysholm scoring gives more points to pain and instability as compared to other items in the questionnaire in the scale. In contrast IKDC grading gives equal importance to all the parameters in the documentation form. Moreover, IKDC has high criterion-related validity for knee injury patients than Lysholm score^28^. In the present study, Lysholm scoring was done for future study comparisons.

In our study, the stress testing with kneeling was conducted for PCL laxity as described by Jung *et al*^29^. In their study of comparison of five different techniques to measure posterior ligament insufficiency, stress testing with active hamstring produced the same results as with Telos stress device in 90 degrees of flexion at knee. Stress radiographs are shown to have better results as compared to arthrometric evaluation using KT 1000 in quantifying the posterior tibial translation^30^. Grade II PCL laxity was observed in seven patients at last follow up however the IKDC grades and Lysholm scores were not affected significantly.

This study had several limitations. The retrospective nature of the study inherited selection bias. The sample size was small and there was inclusion of other associated bony and ligamentous injuries. The duration of follow up was variable ranging from 12 months to 48 months. However, previous studies have shown that the functional results attain a plateau after one year^12^. Therefore, the patients with one year follow up were also included in the study. The strength of the study was in the use of patient specific validated tool like IKDC to assess the functional results and stress radiographs to document the PCL laxity.

## Conclusion

Lag screw fixation of PCL tibial bony avulsion produces acceptable clinical results after stable fixation. The posteromedial approach allows good exposure for screw fixation with minimal dissection. Early rehabilitation instead of cast immobilisation achieves good range of motion and prevents arthrofibrosis.

### Ethical Approval:

The study complies with current ethical considerations. The study protocol conforms to the Ethical Guidelines of the 1975 Declaration of Helsinki.

### Consent:

Written informed consent was obtained from each patient included in the study.

### Conflict of Interests:

There is no conflict of interests to be declared.
